# COVID-19 in the US-affiliated Pacific Islands: A timeline of events and lessons learned from March 2020–November 2022

**DOI:** 10.1371/journal.pgph.0002052

**Published:** 2023-08-16

**Authors:** Haley L. Cash McGinley, W. Thane Hancock, Stephanie Kern-Allely, Melissa Jenssen, Emi Chutaro, Janet Camacho, Pedro Judicpa, Kazuhiro Okumura, Nick Muñoz, Oluwatomiloba M. Ademokun, Richard Brostrom

**Affiliations:** 1 Pacific Island Health Officers’ Association, Honolulu, Hawai‘i, United States of America; 2 Career Epidemiology Field Officer Program, Division of State and Local Readiness, Office of Readiness and Response, United States Centers for Disease Control and Prevention, Hagåtña, Guam; 3 Administration for Strategic Preparedness and Response, U.S. Department of Health & Human Services (HHS), Washington, DC, United States of America; 4 Immunization Operations and Services Branch, United States Centers for Disease Control and Prevention, Hågatña, Guam; 5 Office of Readiness and Response, Division of State and Local Readiness, United States Centers for Disease Control and Prevention, Atlanta, Georgia, United States of America; 6 Division of Tuberculosis, United States Centers for Disease Control and Prevention, Honolulu, Hawai‘i, United States of America; Johns Hopkins Center for Health Security: Johns Hopkins University Center for Health Security, UNITED STATES

## Abstract

The US-Affiliated Pacific Islands (USAPIs) experience many health disparities, including high rates of non-communicable disease and limited health resources, making them particularly vulnerable when SARS-CoV-2 began circulating globally in early 2020. Therefore, many USAPIs closed their borders early during the COVID-19 pandemic to give them more time to prepare for community transmission. Routine virtual meetings were established and maintained throughout the pandemic to support preparedness and response efforts and to share information among USAPIs and support partners. Data collected from these regular virtual meetings were gathered and disseminated through routine regional situational reports. These situational reports from March 27, 2020 to November 25, 2022 were reviewed to develop a quantitative dataset with qualitative notes that were used to summarize the COVID-19 response in the USAPIs. The initial surges of COVID-19 in the USAPIs ranged from August 2020 in Guam to August 2022 in the Federated States of Micronesia. This prolonged time between initial surges in the region was due to varying approaches regarding travel requirements, including fully closed borders, repatriation efforts requiring pre-travel quarantine and testing, quarantine requirements upon arrival only, and vaccine mandates. Delaying community transmission allowed USAPIs to establish testing capacity, immunize large proportions of their populations, and use novel COVID-19 therapeutics to reduce severe disease and mortality. Other essential components to support the USAPI regional COVID-19 response efforts included strong partnership and collaboration, regional information sharing and communication efforts, and trust in health leadership among community members. Valuable lessons learned from the USAPIs during the COVID-19 pandemic can be used to continue to strengthen systems within the region and better prepare for future public health emergencies.

## Introduction

The novel coronavirus SARS-CoV-2 was first identified in December 2019 in Wuhan, China, and by April 2020, over 2 million cases of COVID-19 were reported worldwide in 203 countries [1]. SARS-CoV-2 eventually mutated, and different variants that changed the virus’s transmissibility and virulence began circulating globally. Of significance, the Delta variant became the variant of concern worldwide in May 2021, and the Omicron variant became the variant of concern worldwide in November 2021 [2]. The original Omicron subvariant BA.1 was then replaced by the Omicron subvariant BA.2 in April 2022 as the variant of concern [3].

The COVID-19 pandemic was different in the US-Affiliated Pacific Islands (USAPIs) compared to the U.S. for many reasons, one being their unique geography and small populations. The USAPIs are made up of three U.S. territories (American Samoa, Commonwealth of the Northern Mariana Islands [CNMI], and Guam) and three freely associated states (FASes) that have a unique compact agreement with the U.S. (Federated States of Micronesia [FSM], Republic of Palau, and Republic of the Marshall Islands [RMI]) [4]. The FSM has four geographically separated and culturally unique states (Chuuk, Kosrae, Pohnpei, and Yap). The USAPIs comprise hundreds of islands spanning a geographic region of about 3 million square miles across the Pacific Ocean.

The USAPIs are home to about 500,000 residents in total. According to current World Bank estimates, the population of these six USAPIs ranges from 18,092 in Palau to 168,783 in Guam [5]. Most USAPI residents live on more populated main islands, though about 10% of the overall USAPI population resides on remote outer islands and atolls, especially in FSM and RMI, where the outer island populations make up 35% of the population in Yap, 35% in Chuuk, 26% in RMI, and 4% in Pohnpei (there are no outer islands in Kosrae) [6, 7].

In addition to unique geography, the USAPIs experience many health disparities and have lower life expectancies compared to the U.S., driven mainly by limited health resources and less health spending per capita than the U.S. [8]. One of the most significant health concerns in the USAPIs is a high burden of non-communicable diseases (NCDs) with some of the highest prevalence of obesity and diabetes in the world [9–18]. A regional state of health emergency due to the epidemic of NCDs in the USAPIs was declared in 2010 [19]. This high NCD burden, combined with limited hospital capacity in the USAPIs, caused much concern about SARS-CoV-2 transmission in the region early during the COVID-19 pandemic.

Another reason that there was concern about SARS-CoV-2 transmission within the USAPIs early in the pandemic were the COVID-19 disparities that were observed among Pacific Islanders in the U.S., as highlighted by a Hawai’i study that found that Pacific Islanders residing in Hawai’i had a case rate 7.5 times higher than non-Pacific Islanders living in Hawai’i [20]. Accordingly, the age-adjusted COVID-19 mortality rate among Pacific Islanders in the U.S. was three times higher than whites in the U.S., although rates differed greatly between Pacific Islander ethnicities [21]. For example, one study conducted early in the pandemic found that the COVID-19 mortality rate among Marshallese living in Arkansas was 65 times higher than whites from the same counties [22].

Although there are many differences between the USAPIs and the U.S., the USAPIs receive support from various U.S. federal agencies under the U.S. Department of Health and Human Services (HHS), including the Administration for Strategic Preparedness and Response (ASPR), Centers for Disease Control and Prevention (CDC), Substance Abuse and Mental Health Services Administration (SAMHSA), and Health Resources and Services Administration (HRSA). This support from U.S. federal agencies is often through domestic public health programs due to their special association with the U.S. Additionally, the six USAPIs also receive support from regional and international partner organizations such as the Pacific Island Health Officers’ Association (PIHOA), the Pacific Community (SPC), United Nations Children’s Fund (UNICEF), Asian Development Bank, World Bank, and the World Health Organization (WHO). Coordination of these partner agencies is critical, especially during an emergency response such as the COVID-19 pandemic [23].

It was crucial that regional cooperation and communication was established early on through the implementation of routine one-on-one virtual meetings between each USAPI (including each FSM state) and key partners, complemented by two regional virtual meetings for USAPI partner coordination and USAPI-wide information-sharing and networking. These virtual meetings, in turn, also fed into WHO’s wider Pacific Joint Incident Management Team (JIMT) partner coordination virtual meetings and PIHOA’s monthly USAPI health leadership virtual meetings. These efforts helped ensure that accurate and up-to-date information was shared between technical partners and the USAPIs to have timely, coordinated responses that met their needs as they arose. Information from these virtual meetings also informed critical advocacy work with the US HHS, White House, and international partners, and provided a platform for peer sharing among the USAPIs, including lessons learned that informed ongoing response work across the region and within the USAPIs as the pandemic progressed.

In parallel to these ongoing virtual meetings, routine USAPI regional situational reports were developed by PIHOA and disseminated widely throughout the pandemic [23, 24]. Routine USAPI COVID-19 situational reports included data from all USAPIs on reported COVID-19 cases, deaths, hospitalizations, testing throughput and supplies, travel restrictions and entry protocols, community mitigation measures, medical surge preparedness, inventory and administration of therapeutics, and vaccine administration. These data used in the situational reports were gathered from routine one-on-one virtual meetings with the USAPIs, locally produced reports from the USAPIs, and public online data dashboards from the USAPIs. The USAPI COVID-19 situational report was produced weekly beginning in March 2020, then shifted to a biweekly report beginning May 20, 2022, and continued until September 23, 2022, when monthly reports were developed until November 25, 2022.

The purpose of this paper is to provide a summary of the COVID-19 pandemic in the USAPIs and highlight key lessons learned through a review of these regional situational reports from March 2020 to November 2022.

## Materials and methods

### Ethics statement

This research involved analysis of secondary data and did not involve human participants.

### Data collection and analysis

**Fig 1:**
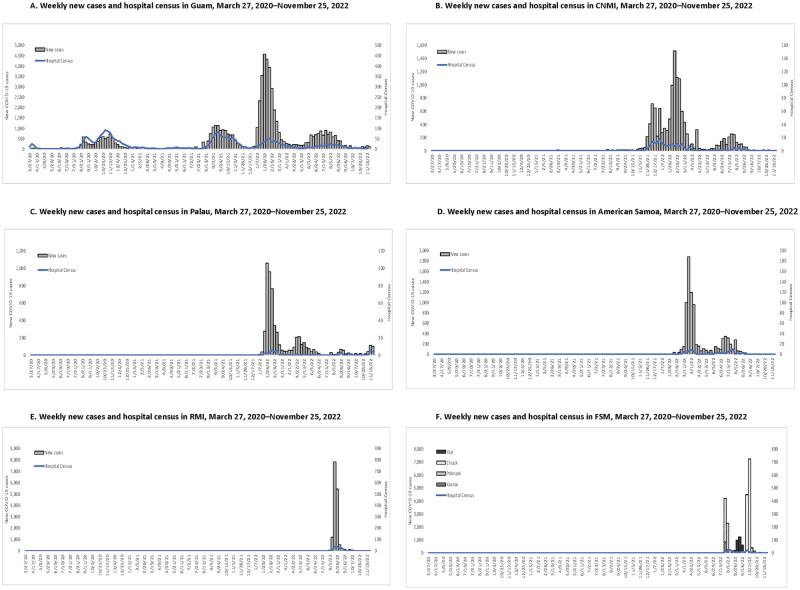
**A**. Weekly new cases and hospital census in Guam, 3/27/20-11/25/22, **B**. Weekly new cases and hospital census in CNMI, 3/27/20-11/25/22, **C**. Weekly new cases and hospital census in Palau, 3/27/20-11/25/22, **D**. Weekly new cases and hospital census in American Samoa, 3/27/20-11/25/22, **E**. Weekly new cases and hospital census in RMI, 3/27/20-11/25/22, **F**. Weekly new cases and hospital census in FSM, 3/27/20-11/25/22.

Regional situational reports (containing quantitative and qualitative details) publicly available on the PIHOA website were reviewed from March 27, 2020, to November 25, 2022, to create a quantitative dataset, and qualitative details were collected and noted during this review [25]. This timeframe was selected based on the availability of regional situational reports. Additionally, jurisdiction-specific situational reports were reviewed from May 20, 2022–November 25, 2022, to collect data on weekly case counts and hospital census to develop accurate epidemiological curves presented in Fig 1. When outliers or inconsistencies appeared in the dataset, numbers or details from the situational reports were cross-checked with locally produced reports and rectified if necessary.

Variables in the dataset included the number of new cases reported each week, the number of new deaths reported each week, the number of current hospitalizations recorded each week, nucleic acid amplification test (NAAT) capacity (yes/no), antigen testing capacity (yes/no), border closure status (closed borders, in-country quarantine requirement, repatriation protocols in place with pre-travel quarantine and testing requirements, vaccine mandate only, or open borders), number of therapeutic doses available on-island, number of therapeutic doses reported being used each week, and number of individuals partially vaccinated, fully vaccinated, and receiving an additional dose. Rates were calculated using denominators of the total population from the most recent U.S. Census data in American Samoa (2020), CNMI (2020), Guam (2020), and Palau (2015). In FSM and RMI, denominators were used based on local projections due to a lack of recent Census data and significant changes to these populations due to emigration. Qualitative notes were taken upon review of all qualitative information available within the situational reports to note details on challenges throughout the pandemic, travel protocols, and vaccine eligibility. These qualitative data were used to summarize lessons learned and support the discussion. Descriptive analysis was performed using SPSS v26.

Strains of SARS-CoV-2 responsible for COVID-19 surges were identified using the U.S. National SARS-CoV-2 Strain Surveillance System [26]. The USAPIs routinely submitted samples for sequencing that were recorded in this system.

## Results

### COVID-19 cases and hospitalizations

The first cases of COVID-19 in Guam were reported in March 2020, and weekly case counts and hospital census remained low until there was a surge from August to December 2020 (Fig 1A). Weekly case counts were low compared to subsequent surges peaking at 474 cases per 100,000 population, though the hospital census was relatively high given the weekly case counts and reached an all-time high of 60 hospitalizations per 100,000 population. A second surge from the Delta variant occurred in Guam from August to December 2021. This surge included higher weekly case counts than the initial surge peaking at 729 per 100,000 population, though the hospital census was similar to the initial surge with a peak of 53 hospitalizations per 100,000 population. Both the original surge and Delta surge in Guam lasted about five months each. A third surge due to the Omicron variant occurred from January to March 2022, in which much higher weekly case counts were reported peaking at 2,972 cases per 100,000 population. However, the hospital census remained relatively low compared to the two prior surges, peaking at 34 hospitalizations per 100,000 population. This third surge was also shorter, lasting about three months. A fourth surge due to Omicron subvariant BA.2 occurred from June to August 2022, in which weekly case counts were similar to the initial surge and Delta surge peaking at 584 cases per 100,000 per population however, the hospitalization census was lower than the three other surges peaking at 13 hospitalizations per 100,000 population.

A small number of community cases of COVID-19 in CNMI were detected in March 2020, but CNMI successfully eradicated this initial transmission through strict lockdown measures. The first significant community transmission of SARS-CoV-2 in CNMI occurred in October 2021 (Fig 1B). In December 2021, consecutive surges from the Delta variant followed by the Omicron variant resulted in cocirculation of both variants. Weekly case counts were higher during the Omicron surge peaking at 3,195 cases per 100,000 population compared to 1,504 cases per 100,000 population during the Delta surge. However, the hospital census was higher during the Delta surge, peaking at 38 hospitalizations per 100,000 compared to 21 hospitalizations per 100,000 population during the Omicron surge. A third surge took place from June to August 2022 due to Omicron subvariant BA.2. This third surge had relatively low case counts and low hospital census peaking at 543 cases per 100,000 population and 11 hospitalizations per 100,000 population, respectively.

Palau identified their first cases of COVID-19 among travelers beginning in August 2021 after quarantine requirements were dropped; however, community transmission of SARS-CoV-2 was not detected until January 2022 when the Omicron variant caused the first surge (Fig 1C). The first surge in Palau was brief, lasting less than three months, though weekly case incidence was high, peaking at 6,005 cases per 100,000 population with a relatively low hospital census, peaking at 34 hospitalizations per 100,000 population. The second surge due to Omicron subvariant BA.2 followed shortly after the initial Omicron surge beginning in April 2022 and lasting only about two months. During this second surge, the weekly case incidence peaked at 1,212 cases per 100,000 population, and the hospital census peaked at six hospitalizations per 100,000 population.

In American Samoa, community transmission of SARS-CoV-2 was not detected until February 2022, when cases of the Omicron variant among quarantine staff workers infected individuals in the community (Fig 1D). This Omicron surge in American Samoa was brief, lasting only about one month, with a high weekly case incidence peaking at 3,784 cases per 100,000 population, and relatively few hospitalizations being reported, peaking at 18 hospitalizations per 100,000 population. Shortly after, American Samoa experienced a second surge due to Omicron subvariant BA.2, which occurred from June to August 2022. Weekly case incidence was much lower than the first surge, peaking at 690 cases per 100,000 population. However, the hospital census was similar during the second surge peaking at 20 hospitalizations per 100,000 population.

In RMI, community transmission of SARS-CoV-2 was not detected until August 2022, when travelers with SARS-CoV-2 in quarantine infected quarantine workers, leading to community transmission (Fig 1E). The initial surge of COVID-19 was due to Omicron subvariant BA.5. It was brief, lasting approximately two weeks. Weekly case incidence peaked at 13,223 per 100,000 population, though hospital census remained relatively low given these high case counts, peaking at 64 hospitalizations per 100,000 population.

In FSM, community transmission of SAR-CoV-2 was first detected in the states of Pohnpei and Kosrae in July 2022, followed by Yap in August 2022 and Chuuk in September 2022. The initial surges in each state were due to Omicron subvariant BA.5, each lasting approximately two weeks. All community transmission in FSM initially occurred due to spillover from in-country quarantine facilities. Case incidence in FSM peaked during Chuuk’s surge reaching 7,017 cases per 100,000 population. The hospitalization census peaked during the simultaneous Pohnpei and Kosrae surges at 26 hospitalizations per 100,000 population.

### Testing and clinical capacity

**Fig 2:**
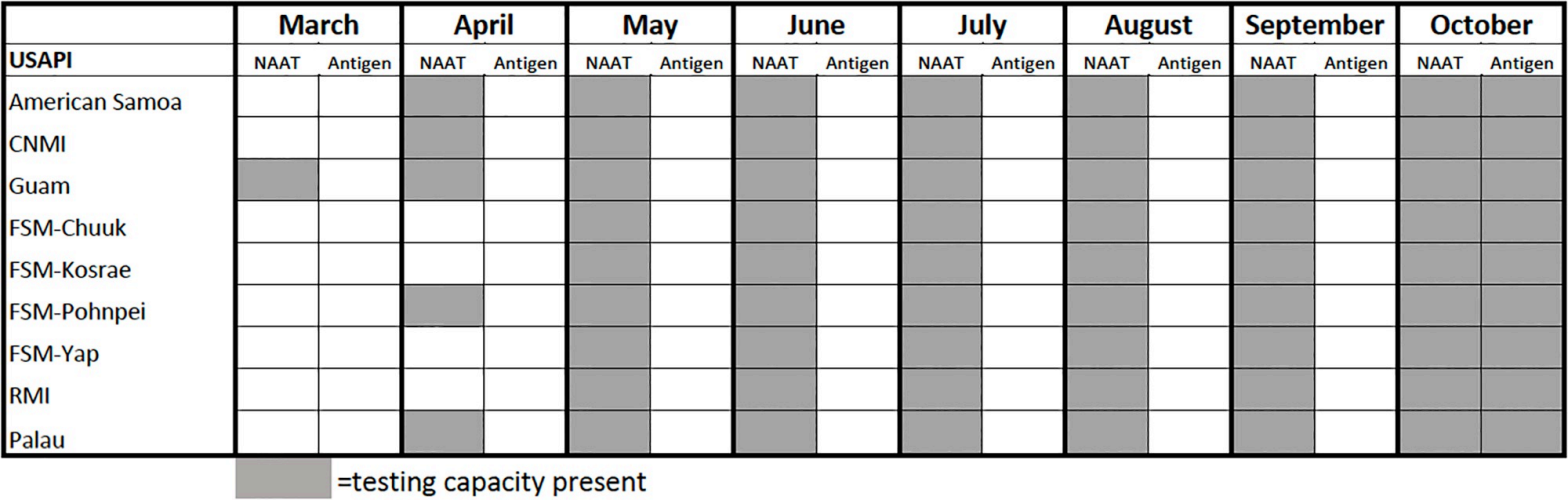
Testing capacity in the USAPI, March-October 2020.

At the beginning of the pandemic in March 2020, only Guam had the capacity to test for SARS-CoV-2 via NAAT (Fig 2). By April 2020, American Samoa, CNMI, Guam, Pohnpei, and Palau had NAAT capacity for the detection of SARS-CoV-2. Chuuk, Kosrae, Yap, and RMI gained their NAAT capacity for SARS-CoV-2 detection in May 2020. SARS-CoV-2 antigen testing capacity was present in all USAPI in October 2020. Free testing was available in all USAPIs once testing capacity was present. Testing availability was limited early in the pandemic due to shortages of testing supplies but was widely available later in the pandemic. By late 2022 there was also widespread availability of home tests. The shift to home testing likely decreased case detection later in the pandemic.

Early situational reports highlighted that USAPIs lacked the appropriate equipment to provide supplemental oxygen therapy to critical COVID-19 patients (e.g., high-flow nasal cannulas and ventilators) as well as clinical staff trained to manage them. Additionally, many USAPIs had inadequate oxygen generation and storage capacity and lacked supplies to deliver oxygen to patients. Early situational reports also highlighted a lack of personal protective equipment, essential medications (such as acetaminophen and ibuprofen), and the inventory management systems to manage these supplies to prevent critical stock-outs.

### Travel requirements and border closures

**Fig 3:**
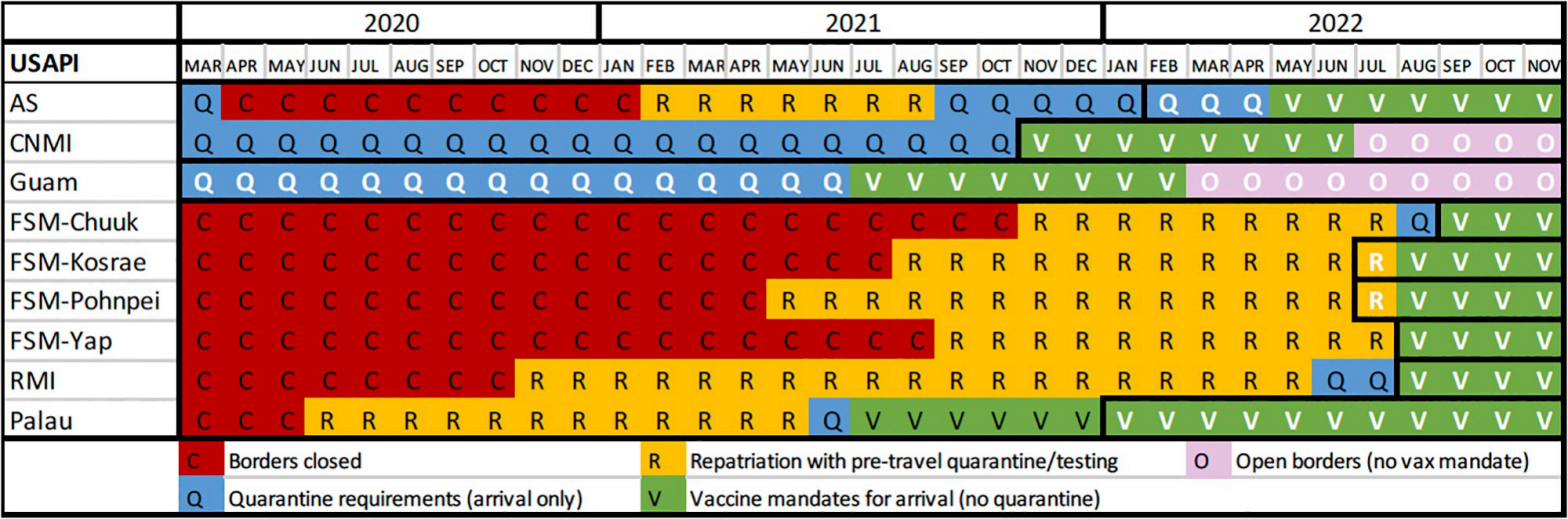
Travel requirements and border closures among the USAPI, March 2020-November 2022*. *White letters indicate widespread community transmission.

In March 2020, all USAPIs but American Samoa, CNMI, and Guam closed their borders to travelers, with American Samoa closing their borders in April 2020 (Fig 3). Only Guam and CNMI kept their borders open throughout the pandemic, though both implemented mandatory quarantine measures much of this time. Guam and CNMI implemented strict quarantine measures early on, requiring all travelers to complete a 14-day facility quarantine upon arrival. These measures were relaxed in Guam in May 2020 to allow for home quarantine with slight modifications over the next 14 months until Guam switched to a vaccine mandate only in July 2021. This vaccine mandate was dropped in Guam in March 2022, allowing for regular travel in and out of Guam. Guam was the only USAPI with consistent community transmission throughout the pandemic. CNMI also relaxed their protocols in May 2020 but kept more rigid quarantine protocols compared to Guam, requiring that all travelers complete a 5-day facility quarantine upon arrival with a day 5 test until November 2021 when CNMI switched to a vaccine mandate only with no mandatory quarantine which was followed by their first COVID-19 surge. CNMI then dropped its vaccine mandate in July 2022.

Palau, RMI, American Samoa, Pohnpei, Kosrae, Yap, and Chuuk implemented repatriation measures beginning in June 2020, November 2020, February 2021, May 2021, August 2021, September 2021, and November 2021, respectively. These repatriation protocols required mandatory facility quarantine before departure in either Guam or Honolulu, then again in-country while undergoing several tests during this process. The number of days required for pre- and post-departure quarantine varied by jurisdiction and changed throughout the pandemic ranging from 3 to 21 days.

Palau retained strict repatriation protocols with pre- and post-departure quarantine and testing until June 2021, when they removed the pre-departure quarantine in Guam and only required in-country facility quarantine for a brief period until they switched to a vaccine mandate only with testing requirements in July 2021. This prevented community transmission until January 2022 due to the Omicron variant. This vaccine mandate is still in place in Palau as of November 25, 2022.

American Samoa removed its pre-departure quarantine in Honolulu in September 2021, switching to only requiring pre-departure testing and a facility quarantine in-country upon arrival with additional testing. This protocol increased the number of cases detected in quarantine in American Samoa and prevented spillover into the community until February 2022. American Samoa shifted to a vaccine mandate in May 2022 that is still in place as of November 25, 2022.

FSM maintained strict repatriation protocols throughout most of the pandemic with pre-departure facility quarantine requirements in Guam (the number of days required was gradually reduced over time). FSM planned to remove quarantine travel requirements on August 1, 2022; however, transmission occurred shortly before this date in Pohnpei and Kosrae when there was spillover from in-country quarantine facilities. FSM switched to a vaccine mandate on August 1, 2022, though Chuuk and Yap still implemented state-mandated in-country facility quarantine requirements after this date until community transmission was detected in Yap in mid-August and Chuuk in late September. Community transmission in Yap and Chuuk was due to spillover from in-country quarantine facilities. FSM still maintains a vaccine mandate to enter the country as of November 25, 2022.

RMI maintained strict repatriation protocols until they detected community transmission from in-country quarantine spillover in August 2022. There was required pre-departure facility quarantine in Honolulu until June 2022, when RMI shifted to a pre-departure test only (no Honolulu facility quarantine), then 14 days of in-country quarantine with testing. Once community transmission was detected, RMI shifted to a vaccine mandate that is still in place as of November 25, 2022.

### Vaccine eligibility

**Fig 4:**
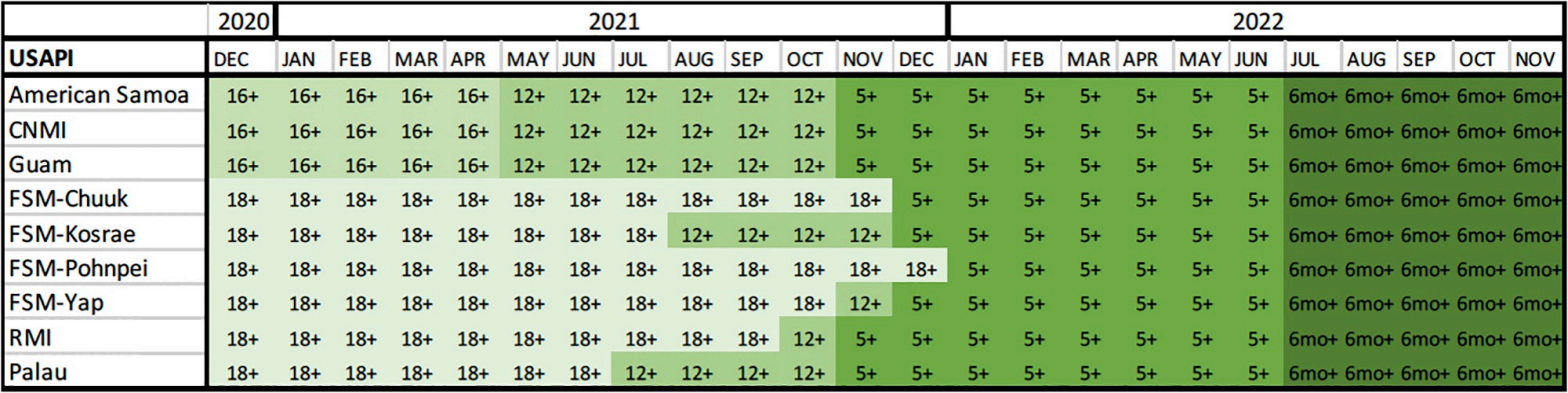
Vaccine eligibility (by EUA/FDA approvals and use on-island) among USAPI, December 2020-November 2022.

Both Pfizer and Moderna vaccines became available to the USAPI in December 2020; however, only American Samoa, CNMI, and Guam were able to use Pfizer early on (the initial U.S. Food and Drug Administration [FDA] issued Emergency Use Authorization [EUA] for the Pfizer vaccine for those 16 years and older) due to initial ultra-cold temperature storage requirements of -70°C [27] (Fig 4). Therefore, eligibility was 16 and older in the territories, whereas eligibility in the FAS was 18 and older initially because they could only use Moderna, which had storage requirements between -25° to -15°C and an FDA EUA for those 18 and older [28]. Eligibility dropped to 12 and older in American Samoa, CNMI, and Guam upon Pfizer FDA EUA for adolescents (12–15 years old) in May 2021. Palau expanded eligibility to 12 and older in July 2021, followed by Kosrae in August 2021, RMI in October 2021, and Yap in November 2021, when Pfizer temperature storage requirements changed. Upon the Pfizer FDA EUA for 5–11-year-olds in November 2021, eligibility expanded to 5 and older in all USAPI but FSM, who delayed 5-11-year-old vaccination efforts, thus delaying eligibility to December 2021 in Chuuk, Kosrae, and Yap, and January 2022 in Pohnpei. All USAPI expanded eligibility to 6 months and older in July 2022 upon expanded EUAs for both Moderna (Moderna FDA EUA was expanded from 18 and older to 6 months and older in June 2022) and Pfizer (Pfizer FDA EUA was expanded to 6 months and older in June 2022) vaccines.

It should also be noted that the Janssen COVID-19 vaccine received FDA EUA for use in those 18 and older in February 2021 [29]. This vaccine had a storage requirement of 2°C to 8°C and required one dose (as opposed to the Pfizer and Moderna vaccines that required two doses). Due to the non-frozen storage requirements and single-dose administration, this vaccine was utilized in many outer islands of the USAPI.

### Vaccine distribution

**Fig 5:**
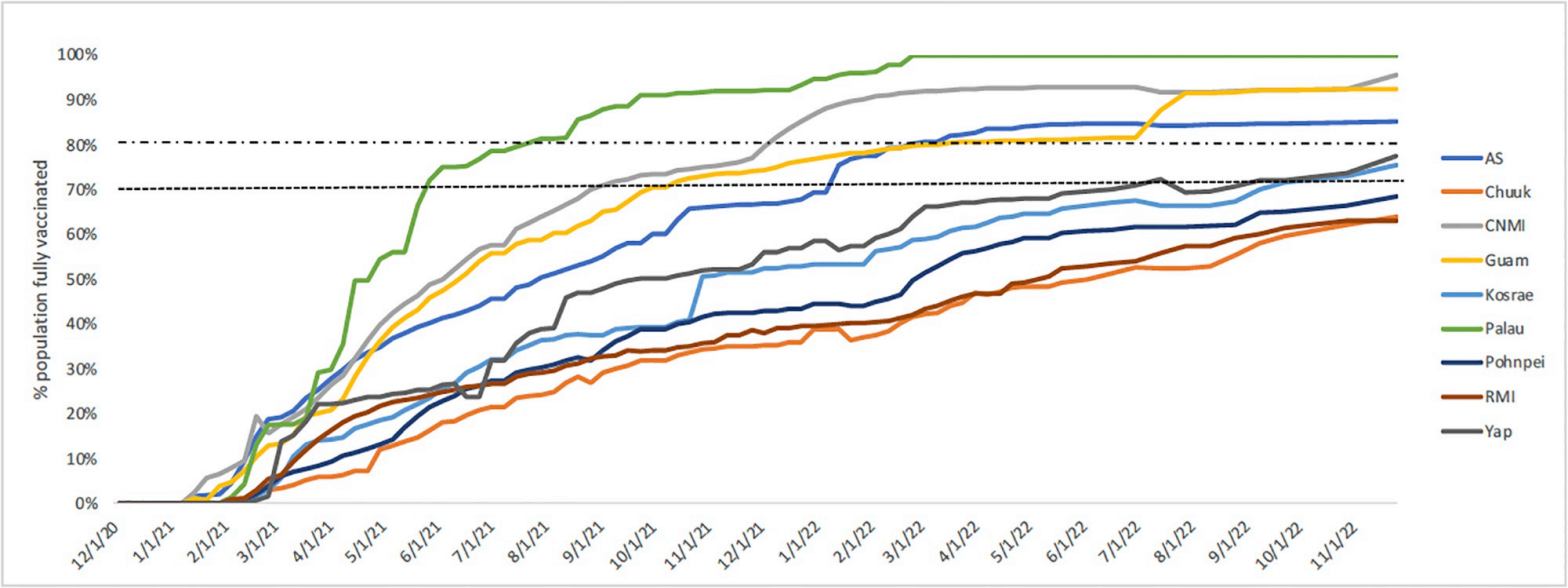
Percent of the population fully vaccinated by USAPI, 12/1/20-11/25/22.

Palau was the first USAPI to fully vaccinate at least 70% of its total population in May 2021, followed by CNMI, Guam, American Samoa, Yap, and Kosrae (Fig 5). As of November 25, 2022, the current percent of the total population fully vaccinated ranges widely in the USAPI at >99.0% (Palau), 95.7% (CNMI), 92.5% (Guam), 85.1% (AS), 77.5% (Yap), 75.6% (Kosrae), 68.4% (Pohnpei), 64.0% (Chuuk), and 63.2% (RMI).

### Additional dose distribution

**Fig 6:**
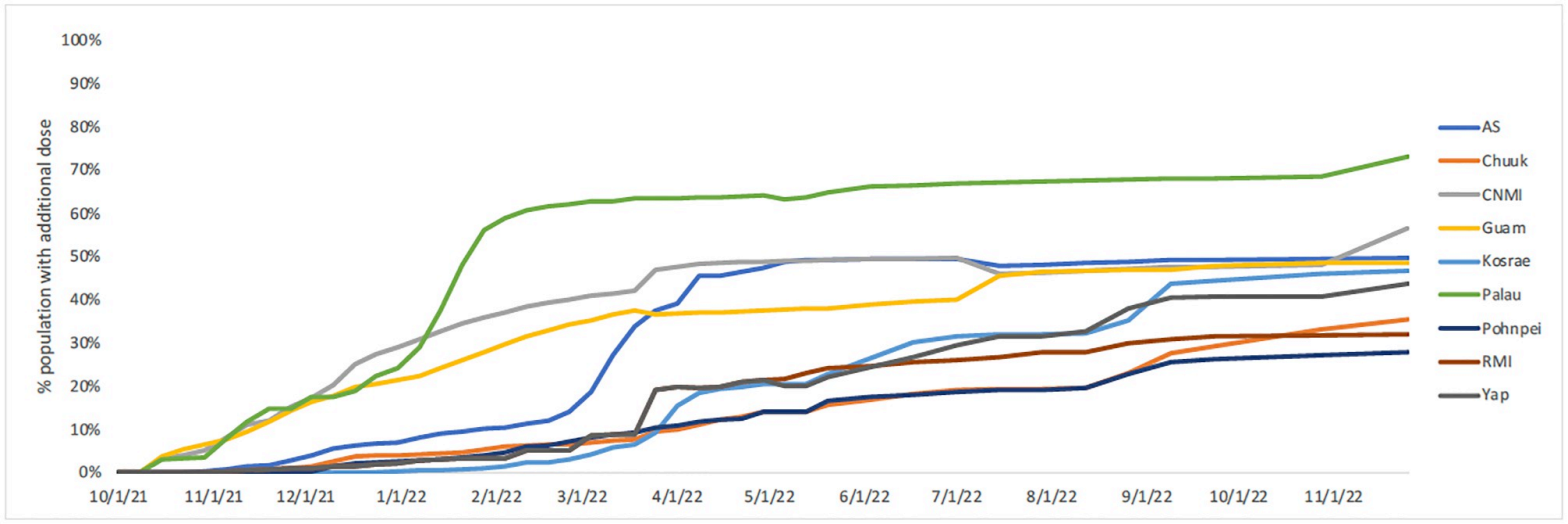
Percent of population with additional vaccine dose, by USAPI, 10/1/21-11/25/22.

Additional doses (third doses and boosters) started being administered in the USAPI in October 2021. Administration was steady in Guam, CNMI, and Palau until community transmission was detected in Palau; then, there was a high uptake of additional doses, reaching over 50% in January 2022 (Fig 6). The remaining USAPI had a slower rollout of additional doses, though there was a similar rapid uptake in American Samoa in March 2022 after community transmission was detected. All four states of FSM experienced modest upticks in additional dose administration after community transmission was detected from August to September 2022. As of November 25, 2022, the current percent of the total population with additional doses ranged widely in the USAPI at 73.0% (Palau), 56.4% (CNMI), 49.5% (AS), 48.5% (Guam), 46.7% (Kosrae), 43.6% (Yap), 35.4% (Chuuk), 31.9% (RMI), and 27.8% (Pohnpei).

### Mortality and therapeutic use

COVID-19 therapeutic (monoclonal antibodies and antivirals) use was reported in the USAPIs beginning in October 2021, and a total of 19,760 therapeutics were administered in the USAPIs as of November 25, 2022 (Table 1). Therapeutic use varied greatly across the region ranging from 2.8% of cases treated in CNMI to 42.4% of cases treated in Chuuk. Overall, there were a total of 564 COVID-19 deaths reported in the USAPIs as of November 25, 2022. The case fatality rate ranged from 0.11% in RMI to 0.69% in Guam.

**Table 1:** Mortality and therapeutic use in the USAPI, March 27, 2020–November 25, 2022.

	Total Population	Total # of cases	Total # of deaths	Case Fatality Rate	Total # therapeutic courses administered	Therapeutic use rate
AS	49,710	8,264	34	0.41%	1,528	18.5%
CNMI	47,329	13,220	41	0.31%	372	2.8%
Guam	153,836	59,293	407	0.69%	2,109	3.6%
FSM-Chuuk	48,654	12,223	27	0.22%	5,188	42.4%
FSM-Kosrae	6,616	1,104	3	0.27%	433	39.2%
FSM-Pohnpei	36,196	5,639	23	0.41%	989	17.5%
FSM-Yap	11,377	3,076	5	0.16%	1,089	35.4%
Palau	17,651	5,785	7	0.12%	1,563	27.0%
RMI	59,194	15,541	17	0.11%	6,489	41.8%
**Total**	430,536	124,145	564	0.45%	19,760	15.9%

## Discussion

Due to the USAPIs’ proximity to and travel between East Asia in early 2020 in addition to little to limited clinical resources to manage severe cases, border closures were initiated early in the USAPIs during the COVID-19 pandemic to prevent early SARS-CoV-2 transmission [30]. These efforts to postpone introduction were an effective mechanism to reduce severe disease and death in the USAPIs. Overall, compared to Guam, the case fatality rate was lower in those USAPIs who were able to delay community transmission through border closures. This is especially important given the limited clinical capacity of the USAPIs to handle large numbers of hospitalizations and patients needing complicated procedures and long-term care [31]. By delaying transmission, the USAPIs with border closures had more time to better prepare for response by building laboratory capacity, strengthening clinical capacity, administering vaccinations, and adopting novel COVID-19 therapeutics. However, it should be noted that these border closures came at an economic cost to the USAPIs and other small island developing states [32]. Additionally, there are other factors that may have influenced these varying case fatality rates and therefore direct causation cannot be statistically proven.

Although borders were closed in many USAPIs throughout most of the pandemic, repatriation systems made it possible to safely return individuals such as stranded residents and residents seeking off-island medical care back into their home jurisdictions. Although these repatriation system protocols varied by jurisdiction and changed throughout the pandemic, having a well-maintained repatriation system kept SARS-CoV-2 out of the community while allowing for essential travel in the USAPIs. However, these repatriation systems were resource-intensive, especially with pre-departure quarantine requirements in Honolulu and Guam. These quarantine procedures required many staff to conduct testing, ensure that travelers maintained quarantine, and ensure safe travel from pre-departure quarantine to in-country quarantine. Additionally, these repatriation systems were expensive to maintain due to staffing, quarantine facility costs (oftentimes hotel rooms), and testing. Also, like complete border closures, these repatriation protocols can have financial and psychosocial impacts on stranded residents [33–35]. Over time, these repatriation protocols were relaxed in the USAPIs, eventually resulting in community transmission.

Although border closures and repatriation systems were effective in the USAPI and could potentially be tools for other potential global pandemics, it is still important to have adequate preparedness efforts in place [36]. Early in the pandemic, laboratory capacity was quite limited in the USAPIs, with Guam being the only jurisdiction in the region that was able to test for SARS-CoV-2 in March 2020. It was not until May 2020 that all USAPIs could test for SARS-CoV-2 on island. Prior to expanding testing capacity in the region, samples were being shipped to Guam and Hawai’i for testing, which caused long delays for results and incurred substantial shipping costs. These shipments continued in some USAPIs even after testing capacity was present due to a lack of test kits and necessary reagents. The existence of the PIHOA Regional Lab Specimen Shipping Mechanism and Lab Revolving fund (in operation since 2004) helped overcome early specimen shipping challenges and is an example of how region-wide solutions can be important to address the challenges of individual jurisdictions [37].

Limited laboratory capacity has been an ongoing issue in the USAPIs, and the COVID-19 pandemic further highlighted this gap [38]. This was exemplified through test supply procurement and shipping challenges throughout the pandemic. Many USAPIs had difficulty procuring test supplies due to the relatively small orders requested compared to larger nations. Additionally, local procurement processes that require complex protocols and many levels of approval made it difficult to procure supplies in a timely manner. Although it in important to build in checks to ensure proper use of government funds, the inflexibility of these systems make procurement for emergencies challenging. Therefore, the USAPIs relied heavily on partners such as the CDC, ASPR, Department of Interior (DOI), and WHO to provide testing supplies or fund procurement and shipping of these supplies. Additionally, PIHOA provided valuable regional assistance with advocacy for supplies via several PIHOA Board Resolutions and procurement and of these critical supplies throughout the pandemic. PIHOA used similar mechanisms to help redistribute limited supplies (particularly tests and therapeutics) within the region to meet the resource demand of the staggered USAPI COVID-19 surges. It was important to have strong logistical and financial mechanisms in place to ship items within the USAPIs quickly.

Limited clinical capacity due to inadequate healthcare infrastructure and supplies and insufficient human resources, has been an ongoing challenge in the USAPIs that was also highlighted during the COVID-19 pandemic [39, 40]. During early clinical assessments in the USAPIs, it was discovered that many USAPIs lacked the appropriate equipment to provide supplemental oxygen therapy to critical COVID-19 patients (e.g., high-flow nasal cannulas and ventilators) as well as clinical staff trained to manage them. Additionally, many USAPIs had inadequate oxygen generation and storage capacity and lacked supplies to deliver oxygen to patients. There were substantial efforts from partner agencies to get supplies and virtual training to the USAPIs. Although border closures allowed time to procure supplies and provide training, they also prohibited the ability to provide any on-site support. However, this did present the opportunity to expand existing telemedicine in some USAPIs which has been a priority for many years, which could be beneficial moving forward [41]. Another gap identified in this area was the lack of personal protective equipment, essential medications (such as acetaminophen and ibuprofen), and the inventory management systems to manage these supplies to prevent critical stock-outs.

Closing borders allowed most USAPIs to immunize large percentages of their population before community spread of SARS-CoV-2. The high vaccination rates helped limit severe disease requiring hospitalizations, thus reducing the clinical burden in those USAPIs who were able to prevent the community spread of SARS-CoV-2 until after much of the population was vaccinated [42]. The vaccination efforts in the USAPIs were substantial and required a lot of resources, but there were valuable lessons learned that can be used to strengthen future mass vaccination campaigns and routine vaccination efforts [43]. It should be noted that these vaccination efforts were particularly challenging in those USAPIs with significant outer island populations (FSM, RMI) where the vaccination coverage remains lower than the rest of the region.

COVID-19 therapeutic use was first reported in the USAPIs beginning in October 2021. These therapeutics were provided to the USAPIs by the U.S. government [44], and for FSM and RMI, a sizeable cache of COVID-19 rapid tests and therapeutics was shipped prior to recording their first cases of community transmission. USAPIs that were able to delay community transmission through border closure were able to treat a higher proportion of cases with therapeutics. Rates of COVID-19 therapeutic use in USAPIs with delayed community transmission was largely due to the rapid implementation of community-based “Test to Treat” centers. More than 70 of these centers operated in FSM and RMI, allowing individuals to be quickly tested using rapid antigen tests [45]. For persons with risk factors for severe COVID-19 disease who tested positive, therapeutics were provided on-site. This community-based approach was especially important given the high rates of NCDs and the resource limitations for managing multiple severely ill and infectious patients in USAPI hospitals. This strategy created excellent access to high-throughput testing and treatment centers, which may have contributed to the lower-than-expected COVID-19 case fatality rates observed in FSM and RMI.

Throughout the COVID-19 pandemic, mechanisms such as the weekly one-on-one virtual meetings with the USAPIs, regional virtual meetings, and routinely produced regional situational reports allowed for effective collaboration between local, regional, U.S. federal, and international partners. These open lines of communication were essential to maintain a synergized ongoing response in the region, and many of these new relationships between partners should continue to be supported [23]. PIHOA was founded by and is still directed by the senior health officials of the USAPI and was critical in ensuring an efficient and effective response across the region. The COVID-19 pandemic highlighted the importance of the PIHOA leadership having a unified voice to identify regional initiatives and advocate for support.

Although there was a lot of information extracted from the USAPI regional COVID-19 situational reports, there were limitations to this study. This includes the use of secondary data gathered from the weekly USAPI COVID-19 situational reports. Although these weekly reports were developed using reliable information collected from one-on-one virtual meetings, locally produced reports, and local data dashboards, there could potentially be some reporting errors. Additionally, throughout the pandemic, there were challenges with reliable denominators for the USAPI. For some jurisdictions, Census data were from 2010, and there had been significant population shifts since then, mostly due to immigration and emigration. Therefore, calculated rates could be over or underestimated due to the use of population estimates in some USAPI. Finally, it should be noted that the introduction of widespread home testing using rapid antigen tests in late 2022 may have greatly impacted the number of cases being reported in later COVID-19 surges.

## Conclusions

Overall, border closures were able to delay community transmission of SARS-CoV-2 and allow the USAPIs to better prepare for the management of cases; however, it is essential to continue to strengthen preparedness efforts in the region [46]. Specifically, the COVID-19 pandemic highlighted the need to continue to build laboratory and clinical capacity within the region [47]. Additionally, government procurement systems should be reviewed and streamlined, and exceptions should be considered during emergencies. Another weakness highlighted during this pandemic were local inventory systems which were oftentimes completely lacking or inaccurate. There is a need to support development and improvement of clinical inventory systems to monitor stocks of essential drugs, personal protective equipment, and other critical supplies.

Essential components to support the USAPI regional COVID-19 response efforts included strong partnership and collaboration, regional information sharing and communication efforts, and trust in health leadership among community members [23]. These valuable lessons learned from the USAPIs during the COVID-19 pandemic can be used to continue to strengthen systems within the region and better prepare for future public health emergencies, as well as improve ongoing health initiatives.
